# Heme Oxygenase 1 in the Nervous System: Does It Favor Neuronal Cell Survival or Induce Neurodegeneration?

**DOI:** 10.3390/ijms19082260

**Published:** 2018-08-01

**Authors:** Mariapaola Nitti, Sabrina Piras, Lorenzo Brondolo, Umberto Maria Marinari, Maria Adelaide Pronzato, Anna Lisa Furfaro

**Affiliations:** Department of Experimental Medicine, University of Genoa, Via L.B.Alberti 2, 16132 Genoa, Italy; Mariapaola.Nitti@unige.it (M.N.); piras.sabri@tiscali.it (S.P.); lorenzo.brondolo@gmail.com (L.B.); umm@unige.it (U.M.M.); maidep@unige.it (M.A.P.)

**Keywords:** HO-1, oxidative stress, carbon monoxide, bilirubin, nervous system, neurodegeneration, Alzheimer’s disease, Parkinson’s disease, ischemia/reperfusion injury, traumatic brain injury

## Abstract

Heme oxygenase 1 (HO-1) up-regulation is recognized as a pivotal mechanism of cell adaptation to stress. Under control of different transcription factors but with a prominent role played by Nrf2, HO-1 induction is crucial also in nervous system response to damage. However, several lines of evidence have highlighted that HO-1 expression is associated to neuronal damage and neurodegeneration especially in Alzheimer’s and Parkinson’s diseases. In this review, we summarize the current literature regarding the role of HO-1 in nervous system pointing out different molecular mechanisms possibly responsible for HO-1 up-regulation in nervous system homeostasis and neurodegeneration.

## 1. Introduction

The heme degradation system was discovered in 1968 by Tenhunen and co-workers who described a unique microsomal enzyme identified as heme oxygenase (HO) [1]. Subsequently, in 1986, Maines, and, in 1993, Ewing described two different isoforms of HO of 32–36 kDa, namely HO-1 and HO-2, expressed in mammalian cells [2,3] and later the third isoform (HO-3) was discovered in rats as a pseudogene [4].

Through the biological activities of its metabolites, heme oxygenase exerts cytoprotection [5,6]. Indeed, HO degrades heme groups to carbon monoxide (CO), free ferrous iron (Fe^2+^) and biliverdin [7,8]. From the activity of biliverdin reductase (BVR), biliverdin is converted to bilirubin which is able to scavenge hydroxyl radicals, singlet oxygen and superoxide anions [9] and prevents protein and lipid peroxidation [10,11], then exerting a strong antioxidant [12], anti-apoptotic [6] and anti-inflammatory activity [13]. Moreover, through the modulation of soluble guanylyl cyclase (sGC) and mitogen-activated protein kinase pathway (MAPK), CO exerts anti-apoptotic and anti-inflammatory effects [5,6,14]. In addition, the release of free iron favors the synthesis of the heavy chain of ferritin, which quenches free iron, and the activation of the membrane transporter Fe-ATPase, which permits the cytosolic iron efflux, decreasing the intracellular free Fe^2+^ content and preventing oxidative cell damage due to Fenton reaction [15,16].

The deregulation of HO system has been associated with the pathogenesis of Alzheimer’s disease (AD), Parkinson’s disease (PD), multiple sclerosis, brain ageing, and its involvement has been demonstrated in neurotoxicity and in the progression of neuroinflammation [17,18,19]. Among the different HO isoforms, HO-2 is constitutive and predominantly expressed in the brain [20]. Neurotoxicity is accentuated in HO-2 knock-out mice and the addition of exogenous bilirubin to neuronal cultures exerts neuroprotective effects [21,22,23]. Thus, a cytoprotective role has been recognized for HO-2 in neurons, especially against brain hypoxia, as reviewed in [24].

HO-1, instead, is the inducible form of heme oxygenase. It is present at low levels in most mammalian tissues and is up-regulated by a number of oxidative stimuli as described below [7,25,26], carrying out antioxidant and anti-inflammatory responses. In normal brain, HO-1 protein expression is low and restricted to small groups of neurons and neuroglia [27]. On the contrary, HO-1 mRNA is physiologically detectable with high levels in the hippocampus and cerebellum suggesting the existence of a cellular reserve of HO-1 transcript quickly available for protein synthesis [20,28,29].

We focused on HO-1 as a key molecule involved in nervous system response to damage. Indeed, the role played by HO-1 is highly complex and not completely understood. While it has been clearly demonstrated that HO-1 activation in neurons is strongly protective against oxidative damage and cell death [30,31], it is also evident that its up-regulation is associated to the late phase of neurodegeneration and has been proposed as biomarker of AD [32]. Evidence related to the function of HO-1 in neuronal cells as well as in astrocytes, oligodendrocytes and in microglia has been taken into consideration.

## 2. Molecular Mechanisms of HO-1 Induction

HO-1 gene (HMOX1) is located on chromosome 22q12. It has five exons, four introns and a promoter region with one proximal and two or more distal enhancers [7]. The presence of different binding sequences for many transcription factors such as Nrf2, NF-κB, Hypoxia-inducible factor 1 (HIF1), Activator Protein 1 (AP-1), Activator Protein 2 (AP-2), and metal-, stress- or cadmium-response elements renders HO-1 the downstream target of different transduction pathways. Then, it is responsive to many stimuli such as heavy metals, radiations, reactive oxygen species (ROS), modified lipids, growth factors, and inflammatory cytokines [7,25,33]. As described for other tissues, Nrf2 is recognized as a pivotal regulator of HO-1 induction also in brain and nervous system [34].

Nrf2 is a redox transcription factor involved in the regulation of the cellular redox state. It is responsible for the activation of several antioxidants and phase I and II drug-metabolizing enzymes [35,36,37]. In normal conditions, Nrf2 is retained into the cytoplasm by its negative regulator Keap-1 which induces its ubiquitination and proteasomal degradation [38]. Under oxidative or electrophilic stressors, Keap-1 is modified and Nrf2 moves into the nucleus where dimerizes with small Maf proteins (sMafs), binds to the Antioxidant Response Elements (ARE) sequences and activates the transcription of its target genes, among which HO-1 [39].

Thus, HO-1 expression is induced by many activators but only few negative regulators are known, namely Keap-1, which acts reducing Nrf2 protein levels as explained, and Bach1 [40,41]. Working as a heme-binding protein, Bach1 in resting conditions dimerizes with sMafs and binds to the ARE/Electrophile Responsive Element (EpRE) sequences preventing ARE-dependent gene transcription. Under oxidative conditions or when the concentration of heme groups increases, Bach1 is modified, displaced from ARE sequences and degraded to proteasome, allowing Nrf2 to bind [42,43]. It is important to note that many stressing conditions, particularly oxidative stressors, favor the release of heme groups from different proteins such as myoglobin, cytochromes, peroxidases, which induces Bach1-dependent HO-1 activation as a mechanism of cell adaptation. The consequent generation of biliverdin and bilirubin confers cytoprotection and the co-stimulation of ferritin and iron-transporters prevents iron toxicity [44]. Conversely, it has been shown that HO-1 up-regulation, depending on its intensity and duration, can increase tissue damage manly through heme-derived iron, which favors oxidative stress in mitochondria and other compartments, as further explained below in this review.

Interestingly, at least two promoter polymorphisms have been hypothesized to influence HO-1 gene transcription, exerting a role in different human diseases [45]. Indeed, especially the (GT)n dinucleotide length polymorphism and the single nucleotide polymorphism (SNP) (−413)TT have been proved to reduce the efficiency of HO-1 transcription, increasing susceptibility to oxidative damage, as widely proved in different cardiovascular diseases [45]. Even though only few works have been published with regard to neurodegeneration so far, it has been proved that GT sequences are highly polymorphic in AD and PD subjects, but no correlation with a specific allele has been demonstrated [46]. Moreover, a correlation among the SNP (−413)TT and the development of AD has been pointed out in subjects carrying also the tau (5′ of exon 1)AA polymorphism [47] or in combination with SNPs in liver X receptor β (LXR β) such as (intron2)TT, (intron 5)AA and (intron 7)TT [48]. These results underline the protective effect of HO-1 induction in preventing tau aggregation and oxysterols accumulation.

However, molecular mechanisms involved in HO-1 induction and, possibly, in its dysregulation are still far from being completely understood, for instance as far as the modifications of the machinery driving HO-1 transcription is concerned. In particular, it has been recently proved that Bach1 post-transcriptional modifications, especially ubiquitination, regulate its binding to HO-1 promoter and are involved in the progression of AD [49]. In addition, in non-neuronal cell types, MafK has been proved to be involved in HO-1 repression [50] and MafG in Nrf2 binding to ARE [51] and post-transcriptional modifications such as ubiquitination, sumoylation or acetylation have been postulated in the regulation of sMaf proteins as well [52]. Interestingly, in nervous system, MafK and MafG genetic manipulations have been proved to be selectively involved in ARE-dependent transcriptional abnormalities coincident with neuronal degeneration [53], even though, in this context, the involvement of sMafs in the regulation of Bach1 as well as of Nrf2 binding to ARE is still unknown.

In addition, it seems important to consider the role played by microRNAs (miRs) in this context. In fact, the so-called “redoximiRs” have been identified as crucially involved in different pathological conditions, and different miRs have been proposed to regulate Nrf2, Bach1 and HO-1 in neuronal cells [54]. For instance, has-miR-590-3p contributes to the induction of HO-1, NAD(P)H dehydrogenase [quinone] 1 (NQO1), Glutamate-Cysteine Ligase Modified subunit (GCLM), and Glutamate-Cysteine Ligase Catalitic subunit (GCLC) conferring protection from oxidative damage [55]. The inhibition of miR-153 favors neuron survival during cerebral ischemia/reperfusion injury by up-regulating Nrf2/HO-1 axis [56] and, in the same context, miR-424 enhances Nrf2 activity favoring neuron survival [57,58]. Furthermore, we recently proved that miR-494 favors HO-1 up-regulation independently on Bach1, in a neuroblastoma cell line exposed to oxidative stress [59]. Interestingly, it has also been proved that miRs can be the targets of HO-1 activity, especially of HO-1 derived bilirubin, CO and iron, which have been proved to modulate the expression of different miRs in astrocytes [60], opening a new scenario in understanding HO-1 functions.

## 3. HO-1 Protective Effects in Nervous System

Oxidative stress is recognized as pivotal player in the pathogenesis of many neurodegenerative conditions and it has been widely proved that HO-1 induction due both to genetic manipulation and pharmacological treatments provides stress tolerance and neuroprotection. Indeed, in neuroblastoma cell cultures HO-1 overexpression [30,61] or HO-1 up-regulation induced by plant-derived compounds such as tetrahydroxystilbene glucoside [62] have been proved to be effective in reducing β-amyloid induced oxidative stress and H_2_O_2_ dependent neuronal cell death.

The protective activity has been proved to be due to the generation of the end-products of HO-1 activity, as demonstrated by using ferulic acid which counteracts β-amyloid induced oxidative stress through the increased generation of bilirubin and CO [63]. In this context, we recently showed that HO-1 dependent endogenous generation of bilirubin is responsible for neuroblastoma cell resistance to H_2_O_2_ but the efficiency of HO-1 induction decreases with neuronal differentiation, underlining the idea that neuronal commitment can reduce HO-1 dependent adaptability [64]. Other authors proved that CO inhibits the activation of AMP-activated protein kinase (AMPK) which is implicated in the β-amyloid induced toxicity [65]. Similar results have been obtained considering glutamate toxicity. Indeed, cerebellar granule cells from HO-1 overexpressing transgenic mice are more resistant to glutamate and oxidative injury [31]. The protective role of HO-1 against oxidative neuronal damage has been confirmed by experiment performed on animal models. Indeed, the intrathecal administration of cocaine- and amphetamine-regulated transcript (CART) neuropeptides protects against neurodegeneration induced by β-amyloid microinjection in rat brain [66], and mouse treatment with soybean-derived phytoalexin glyceollin prevents glutamate-induce neuronal damage and improve cognitive function, in both cases through the up-regulation of Nrf2 and HO-1 [67]. In addition, in cortex and hippocampus from rats fed with Nrf2 activator tBHQ, the induction of HO-1 resulted protective against lead-induced oxidative damage [68].

The protective role of HO-1 up-regulation has been also proved as far as the PD is concerned., The administration of Simvastatin, by up-regulating HO-1, exerts neuroprotective effects improving antioxidant responses against 6-hydroxydopamine (6-OHDA)-induced PD in animals [69]. Interestingly, in this context, it has been proved that the neuroprotectant 2′,3′-dihydroxy-4′,6′-dimethoxychalcone (DDC), by up-regulating Nrf2/HO-1 in glial cells, is able to protect dopaminergic neurons against 6-OHDA-induced death, through the crucial contribution of cell-to-cell transmission of CO [70]. Moreover, HO-1 can exert cytoprotection favoring proteasomal degradation of α-synuclein and tau which has been proved to reduce the accumulation of toxic protein aggregates in PD or AD [44,71]. Indeed, the reduction of proteasome activity is involved in the formation of protein aggregates and contributes to the pathogenesis of various neurodegenerative conditions [72]. In human neuroblastoma M17 cells, HO-1 overexpression promotes α-synuclein proteasomal degradation through the generation of iron and CO, and independently on the direct HO-1/α-synuclein interaction or on other non-enzymatic functions of HO-1 [73]. In the same experimental model, the overexpression of HO-1 strongly reduces tau protein levels, by downregulating ERK pathway [61].

Furthermore, the neuroprotective action of HO-1 may be due not only to its antioxidant and anti-inflammatory activity but also to the enhancement of neurotrophic factor generation. Adenovirus-induced HO-1 overexpression in substantia nigra up-regulates Brain-derived neurotrophic factor (BDNF) and Glial cell-derived neurotrophic factor (GDNF) production in dopaminergic neurons and glia, respectively [74,75]. In addition, the involvement of HO-1 downstream products in the modulation of BDNF and GDNF expression in neurons and astrocytes has been demonstrated. Indeed, low concentrations of free-form bilirubin promote GDNF or BDNF induction through the activation of MEK, Akt and NF-κB pathways [76], and CO increases the expression of neurotrophic factors via sGC-PKG-dependent pathway [75]. More recently, HO-1 role in favoring the release of neurotrophic factors has been confirmed in mice subjected to stroke that show better recovery activating BDNF-PI3K/Akt signaling in HO-1 overexpressing hippocampi [77]. Interestingly, it has been recently demonstrated that resveratrol exerts protective activity against LPS-induced cytotoxicity in oligodendrocytes restoring BDNF, GDNF and TGF-β generation, through the activation of Nrf2 and HO-1, highlighting the existence of a HO-1 dependent glioprotective pathway [78].

HO-1 induction has also been proved to be involved in brain defense after ischemia. Indeed, transgenic mice overexpressing HO-1 in neurons exhibit low levels of lipid peroxidation end-products, and enhanced expression of the anti-apoptotic protein bcl-2 after cerebral ischemia [79]. In addition, animal exposure to pterostilbene prevents ischemic brain damage in newborns through the Nrf2-dependent induction of HO-1 [80], and, with the same biological meaning, the protective effect of ischemic preconditioning is abolished in HO-1 gene deleted mice [81].

Moreover, HO-1 up-regulation plays a protective role against traumatic brain injury (TBI) and hemorrhage. In fact, HO-1 expression in glial cells exerts neuroprotection in animal models of TBI [82]. In a mice model of selective HO-1 overexpression in astrocytes, a significant neuroprotection after acute intracerebral hemorrhage has been proved [83,84] and, in the same context, systemic hemin administration reduces blood brain barrier (BBB) damage and improves neurological recovery [85]. Furthermore, animal treatments aiming at HO-1 up-regulation, through the modulation of Nrf2 [86,87] or PPARα [88], confirm HO-1 induction as protective response against post-hemorrhagic neuronal dysfunctions. However, it is important to note that HO-1 up-regulation after brain hemorrhage seems to follow a specific time course, with the early events that can be protective against oxidative stress, whereas late stage overexpression may result in dysfunctions and toxicity [89]. In addition, it has recently been highlighted that HO-1 overexpression in microglia is necessary to attenuate neuronal cell death and maintain cognitive functions in a mouse model of subarachnoid hemorrhage (SAH) [90].

Different studies on microglial cells showed that HO-1 up-regulation, mainly due to Nrf2 activation, has a strong anti-inflammatory effect. This has been proved both in cell cultures and in animal models treated with different natural compounds able to activate Nrf2/HO-1 reducing LPS-induced pro-inflammatory activation [91,92,93] and also reducing oxidative damage [94,95].

Moreover, HO-1 activity seems to play an important role against demyelination. Indeed, in an animal model of cuprizone-induced demyelination, the administration of the flavonoid myricetin improves motor dysfunction through the up-regulation of Nrf2 and HO-1 [96]. Furthermore, in experimental autoimmune encephalomyelitis (EAE), a mouse model of multiple sclerosis, the expression of HO-1 is related to the pathological outcome. Indeed, HO-1 knockout mice show enhanced demyelination, paralysis and mortality. Importantly, the induction of HO-1 obtained with colbalt protoporphyrin IX (CoPPIX) [97], erythropoietin [98], or sulforaphane [99] modulates autoimmune neuroinflammation and is able, also in mice with previously established EAE, to revert paralysis leading to disease regression. However, it is important to note that opposite evidence has been published pointing out that the efficacy of epigallocatechin-3-gallate and glatiramer acetate in the therapy of EAE is in part due to the inhibition of HO-1 [100].

Interestingly, in a mouse model of Guillain-Barré syndrome (GBS), the treatment with dimethyl fumarate strongly prevented autoimmune neuritis favoring macrophages M2 polarization through the up-regulation of Nrf2 and HO-1 [101].

Finally, an important role of HO-1 in reducing neuropathic pain has been recently pointed out, showing that HO-1 up-regulation could elicit potent analgesic effects in part due to the inhibition of spinal microglia activation, as shown in a mouse model of peripheral nerve injury [102] or favoring the antinociceptive effect of morphine in diabetic animals [103]. It has been also shown that the HO-1 dependent reduction of neuropathic pain is due to the specific modulation of MAPKs and Nrf2 and the reduction of oxidative stress and neuroinflammation in prefrontal cortex and hypothalamus [104].

## 4. HO-1 Up-Regulation in Neurodegeneration

In the previous paragraph, we focused on pro-surviving effects elicited by HO-1 in nervous system. However, HO-1 up-regulation has been widely associated with neuronal damage and degeneration as well [105].

It has been hypothesized that HO-1 expression increases with ageing in human normal brain, as the immunoreactivity for HO-1 progressively increases between 3 and 84 years of age both in neurons and neuroglia and in cerebral cortex and hippocampus [106]. However, other studies showed that in non-demented subjects, HO-1 immunoreactivity in neurons and in the temporal cortex is not detectable [107] and HO-1 positivity is limited to normal substantia nigra pars compacta [108]. These results led to the hypothesis that during normal ageing, HO-1 can be up-regulated in specific neuronal populations particularly susceptible to oxidative stress as an adaptive defense mechanism. In this context, we previously showed an age-dependent up-regulation of HO-1 in rat liver tissue, followed by an impairment of HO-1 induction in aged animals [109]. It seems important to note that the neuroprotective effect of HO-1 induction is mainly due to the Nrf2-dependent activation, as widely discussed earlier in this review. In ageing as well, for instance, there is evidence that the Nrf2-dependent HO-1 up-regulation due to the treatment with flavonoids or carotenoids is protective against galactose-induced ageing-like neurodegeneration [110,111] and that voluntary running, through the up-regulation of HO-1, improves age-related cognitive decline in rats [112]. The role played by Nrf2 in ageing and neurodegenerative diseases has been widely reviewed elsewhere and there is a general agreement on the fact that in older organisms there is a decline of Nrf2 activity [113]. However, it remains well established that HO-1 is overexpressed in the brains of patients with AD, mainly in the hippocampus and cerebral cortex, and co-localizes to neurons, neurofibrillary tangles, GFAP-positive astrocytes, choroid plexus epithelial cells, ependyma, corpora amylacea and senile plaques [107,114]. Furthermore, HO-1 is strongly expressed in nigral astroglia and in dopaminergic neuronal Lewy bodies in PD [108]. Moreover, as already mentioned, during acute brain damage and hemorrhage, in which HO-1 up-regulation is mainly associated with tissue protection, HO-1 can be subjected to protracted up-regulation which becomes dangerous for the tissue [89].

It has been postulated that HO-1 up-regulation in glial compartment may promote bioenergetics failure, pro-toxin bioactivation, macroautophagy and corpora amylacea formation, by affecting iron metabolism and mitochondrial activity, as reviewed in [44]. Indeed, transferrin receptor-independent accumulation of iron and mitochondrial electron transport (complex I) deficits may be promoted by the enhanced glial HO-1 activity [115,116,117,118]. In addition, the redox-active glial iron mediates bioactivation of 1-methyl-4-phenyl-1,2,3,6-tetrahydropyridine (MPTP) to the dopaminergic toxin 1-methyl-4-phenylpyridine (MPP^+^), despite the inactivation of monoamine oxidases [119] and that the selective overexpression of HO-1 in astrocytes of transgenic mice induces parkinsonian features [120]. In addition, the therapeutic effect of targeted inhibition of glial HO-1 has been proved both in cell cultures and in transgenic mouse model of AD [121].

However, it is important to report that opposite results have been recently published, proving that HO-1 up-regulation in astrocytic compartment is protective against neurodegeneration, as shown by exposure to the HO-1 inducer CoPPIX which protects astrocytes from β-amyloid toxicity [122] and that specific HO-1 induction in astrocytes may protect dopaminergic neurons preventing PD in MPTP-treated mice [123]. Unfortunately, in these reports, no evidence of the signal pathway involved in HO-1 induction is provided.

Furthermore, another important aspect strongly deregulated in glial compartment due to HO-1 overexpression is the homeostasis of cholesterol. Indeed, following transient transfection of human HO-1 in rat astrocytes, total cholesterol content significantly decreases while oxysterols formation increases [124]. In the same cells, the heme degradation products—iron and CO—stimulate cholesterol efflux via activation of the liver-X receptor (LXR) [125]. It has been proved that, in normal ageing brain, sterol homeostasis is maintained and slightly influenced by glial HO-1 expression which, instead, increases in AD and stimulates cholesterol biosynthesis and oxysterol formation. In particular, in the early phase of the disease, total cholesterol levels remain normal or diminished since glial cholesterol efflux is also induced and exceeds its biosynthesis. Conversely, in late AD, the diffuse neuronal degeneration impairs cholesterol efflux generating a strong increase in free cholesterol which exacerbate β-amyloid deposition and neurodegeneration [44,126].

However, we believe that in this context it is important also to consider that HO-1 induction due to genetic manipulation is likely to produce a strong imbalance in the whole HO-1 dependent molecular pathway. For instance, deregulating the balance between HO-1 activity and iron quenching activity, which is fundamental to prevent iron-dependent toxicity. The correlation between the protein level of HO-1 and the cytoprotective or cytotoxic outcome was demonstrated years ago by using an inducible HO-1 vector in fibroblasts, clearly proving that there is a threshold of HO-1 up-regulation beyond which the generation of free iron becomes toxic [127].

In addition, it can be hypothesized that the neurotoxic effect of HO-1 induction may be consequent to a Nrf2-independent activation. This has been demonstrated in the MPP^+^-induced parkinsonian rat model, where Nrf2 downregulation and HO-1 up-regulation have been observed in association with the loss of nigral dopaminergic neurons [128]. Instead, in a similar experimental model, the Nrf2-dependent activation of HO-1 induced by gintonin [129] or gastrodin [130] is protective against neurodegeneration. Interestingly, it has been reported that brain astrocytes exposed to high glucose concentration undergo apoptosis through the activation of HO-1 dependent on NF-κB and AP-1 [131], supporting the hypothesis of an Nrf2-independent neurotoxic HO-1 activation.

Finally, HO-1 has been proposed as systemic marker in early sporadic AD. Indeed, plasma HO-1 protein levels are significantly decreased in patients with probable sporadic AD [132]. The up-regulation of HO-1 in AD brain can be explained as a consequence of local oxidative stress. Instead, the mechanism responsible for the downregulation of HO-1 in the blood of AD patients remains unclear, even though the existence of a HO-1 suppressor that inhibits HO-1 mRNA levels in the lymphocytes in AD plasma has been proposed [32,133]. However, the results about HO-1 plasma levels in patients with AD are controversial. Another study reports no changes in the serum level of HO-1 in a big cohort of AD patients in comparison to normal elderly control samples, and also reports the increased level of serum HO-1 in Parkinson patients, underlining the existence of different mechanisms involved in the peripheral response to oxidative stress in the two diseases [134]. Moreover, another study reports that in plasma of probable AD patients, both HO-1 and biliverdin reductase (BVR) levels are increased as a consequence of the enhanced oxidative stress, and plasma BVR status, more than HO-1, is proposed as potential biochemical marker for the prediction of AD at the earliest stages of disease [135].

## 5. Conclusions

HO-1 up-regulation represents a powerful mechanism of cell adaptation to stress and its antioxidant, anti-apoptotic and anti-inflammatory properties are mainly due to the biological activity of its metabolic products. However, to obtain cytoprotection, HO-1 up-regulation needs to be followed by a parallel activation of other enzymes such as biliverdin reductase which is crucial to guarantee the generation of bilirubin from biliverdin or heavy chain of ferritin and iron transporters, since HO-1 derived free iron needs to be rapidly quenched.

It is conceivable that one of the factors involved in cytoprotective or cytotoxic outcome of HO-1 up-regulation may be related to the signaling pathways involved in HO-1 induction. Indeed, Nrf2-dependent activation of HO-1 is generally linked to a protective adaptation of neurons and glial cells as well, while the Nrf2-independent activation of HO-1, which often involves AP-1 or NF-κB, seems to exert neurotoxic effects. This is probably due to the ability of Nrf2 to also drive the transcription of other antioxidant and protective genes and proteins involved in iron quenching, chelation and transport.

**Figure 1:**
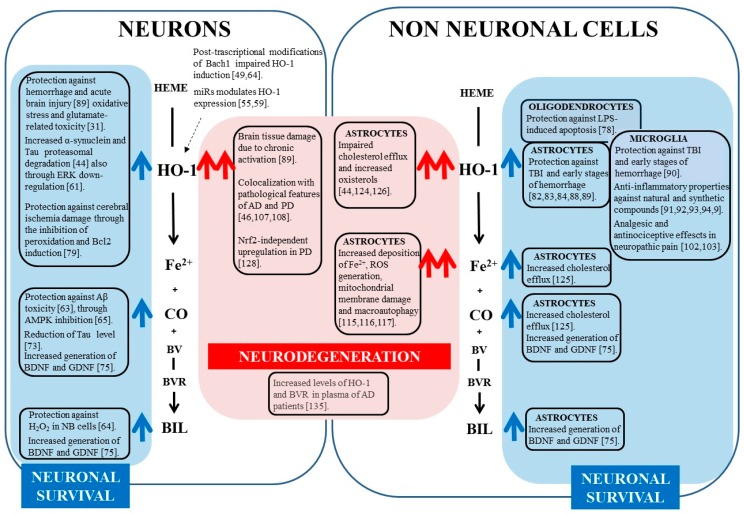
Schematic representation of HO-1 dependent pathway activated in neurons and in the different glial cells. The opposite outcomes (neuronal survival/neurodegeneration) are highlighted and related to the intensity of HO-1 activation and to the amount of free iron and CO generated in neurons and glial cells. The blue arrows indicate the involvement of HO-1 and its metabolic products in neuronal survival; red arrows indicate the involvement of HO-1 and its metabolic products in neurodegeneration; dashed arrow indicates the involvement of miRs in regulating HO-1 expression acting on different molecular targets.

**Figure 2:**
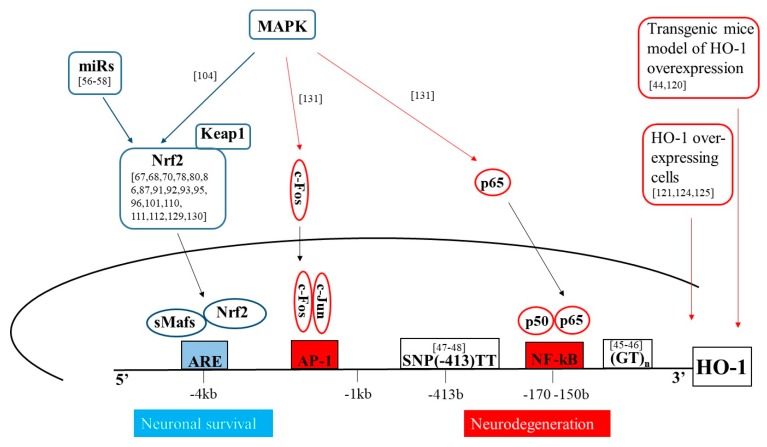
Schematic representation of the main pathways involved in HO-1 mediated neuroprotection or neurodegeneration. The blu arrows and shapes represent signal pathways involved in HO-1 regulation in neuronal survival; the red arrows and shapes represent signal pathways involved in HO-1 regulation in neurodegeneration.

To be protective, HO-1 activity needs to be maintained in a well-defined molecular pathway which involves the generation of heme, its degradation and the ultimate conversion of HO-1-derived products in non-toxic and active compounds. The overall exacerbation of this protective mechanism can increase cell resistance to many stressors, favoring the gain of an aggressive tumor phenotype [136], but the untimely, prolonged or excessive activation of HO-1 can easily produce a deleterious cytopathic effect. In the nervous system, this mechanism becomes even more complex due to cell–cell interactions, characteristic of brain tissue. In fact, the dysregulation of heme degradation pathway through the alteration of iron metabolism and the mitochondrial impairment leads to neurodegeneration not only when it occurs in neurons but also, typically, when it occurs in glial cells. The main concepts have been summarized in Figure 1 and Figure 2 and Table 1.

## Figures and Tables

**Table 1:** Effects of HO-1 induction in neurological diseases.

Disease	Effect	Summary	Experimental Model	Refs
**AD**	Neuroprotection	HO-1 overexpression reduces tau expression and inactivates MAPK cascade.	NB cells	[61]
		HO-1 reduces β-amyloid toxicity through CO generation and AMPK inhibition.	SH-SY5Y NB cells and rat primary neurons	[65]
		HO-1 exerts cytoprotection promoting tau proteasomal degradation.	M17 NB cells	[44,71]
		Tetrahydroxystilbene glucoside up-regulates HO-1 and increases neuronal survival.	HT-22 cells exposed to Aβ	[62]
		CART neuropeptides up-regulate Nrf2/HO-1 axis favoring neuroprotection.	AD rat model (Aβ injection in brain)	[66]
	Neurodegeneration	HO-1 is overexpressed in AD brains and co-localizes to neurons, astrocytes, choroid plexus epithelial cells, ependyma, corpora amylacea, neurofibrillary tangles and senile plaques.		[44,107,114]
		Targeted suppression of glial HO-1 by using HO-1-inhibitors exerts neuroprotection.	APPswe/PS1ΔE9 transgenic mice, rat astrocytes overexpressing HO-1	[121]
		Transient transfection of rat astroglia with human HO-1 cDNA, significantly decreases intracellular cholesterol content and increases oxysterols levels.	Primary neonatal rat astrocytes	[124]
		HO-1 overexpression and its byproducts stimulate cholesterol efflux via activation of liver-x-receptor.	Primary neonatal rat astrocytes	[125]
		HO-1 overexpression in astroglia from AD brains promotes the oxidation of cholesterol to oxysterols.	Primary neonatal rat astrocytes	[44,126]
		Plasma HO-1 protein levels are significantly decreased in patients with probable sporadic AD.	Sporadic AD and MCI Patients	[32,132]
		The activity of the HO-1 suppressor α1-antitrypsin reduces HO-1 expression in AD plasma.	Sporadic AD patients	[133]
		The HO-1/BVR status in plasma is a potential biomarker for the earliest stages of AD.	Plasma from probable AD patients	[135]
**PD**	Neuroprotection	HO-1 overexpression favors α-synuclein proteasomal degradation through the generation of iron and CO.	M17 NB cells	[44,73]
		HO-1 induction exerts a neuroprotective effect in dopaminergic neurons and glia through the enhancement of neurotrophic factor generation.	Parkinsonian rat model	[73,74,75]
		DDC prevents 6-OHDA-induced dopaminergic neuronal death through the up-regulation of glial expression of HO-1.	C57BL/6N mice and primary mesencephalic cultures from rat embryos	[70]
		ATR-I reduces the inflammatory response exerting by inducing HO-1.	Male C57BL6/J mice, BV-2 mouse microglial cells	[92]
		Vinyl sulfone activates Nrf2/HO-1 axis preventing neuroinflammation in microglia and in an animal model of PD.	Male C57Bl/6 mice, BV-2 mouse microglial cells	[95]
		Simvastatin up-regulates HO-1 and increases antioxidant responses in PD.	Mice treated with 6-OHDA and SH-SY5Y cells exposed to 6-HODA	[69]
	Neurodegeneration	HO-1 is overexpressed in nigral astroglia and in dopaminergic neuronal Lewy bodies.		[107,114]
		HO-1 up-regulation is associated with the loss of nigral dopaminergic neurons.	Wistar rats exposed to 1-methyl-4-phenylpyridine (MPP^+^)	[119]
**Neuronal injury/Neurotoxicity**	Neuroprotection	HO-1 protects neurons against oxidative stress-induced injury.	SN56 NB cells	[30]
		HO-1 overexpression protects from glutamate toxicity and H_2_O_2_-induced cell death.	HO-1(−/−) Tg mice	[31]
		Glyceollin increases MAPK/Nrf2/HO-1 pathway and protects against glutamate-induced toxicity.	HT22 cells	[67]
		Ferulic acid mediates neuroprotection through HO-1 up-regulation.	SH-SY5Y NB cells	[63]
		t-BHQ-mediated induction of Nrf2/HO-1 pathway protects against lead neurotoxicity.	SH-SY5Y NB cells, cortex and hippocampus from rat	[68]
		HO-1-derived bilirubin protects neuronal cells from oxidative stress but neuronal differentiation decreases HO-1 induction.	SH-SY5Y NB cells	[64]
		Quercetin increases Nrf2/HO-1 favoring neuroprotection against galactose-induced damage.	D-galactose treated mice	[110]
	Neurodegeneration	Lycopene reduces neuroinflammation and improves cognitive functions in a model of ageing-like neurodegeneration.	D-galactose treated mice	[111]
		Glial HO-1 up-regulation promotes abnormal patterns of iron deposition and mitochondrial insufficiency in different human neurodegenerative disorders.	Rat primary astrocytes and Tg mice over-expressing HO-1	[114,116,117]
**Inflammation**	Neuroprotection	Resveratrol restores GDNF, BDNF and TGF-β production from oligodendrocytes by up-regulating Nrf2/HO-1 axis.	Oligodendrocyte progenitor cells from Wistar rats	[78]
		LPS-induced neuroinflammation is inhibited by Sophoraflavanone G through nuclear translocation of Nrf2 and HO-1 upregulation.	BV2 mouse microglial cells	[91]
		Tryptanthrin protects against LPS-induced inflammation via Nrf2/HO-1 antioxidant signaling.	C57BL/6 mice, BV2 mouse microglial cells	[93]
		Licochalcone E exerts anti-inflammatory and cytoprotective effects activating the Nrf2/HO-1 pathway.	BV2, HEK293T and SH-SY5Y cells.	[94]
		HO-1 and its end-product CO have a protective effect against autoimmune neuroinflammation in experimental autoimmune encephalomyelitis.	C57BL/6 and SJL/J mice, BV2 mouse microglial cells	[97]
		EPO up-regulates endogenous HO-1 and represses immune and inflammatory responses in experimental autoimmune encephalomyelitis.	C57BL/6 mice	[98]
		Sulforaphane, by enhancing Nrf2/HO-1 activity, antagonizes autoimmune inflammation and inhibits EAE development and severity.	C57BL/6 mice	[99]
		Myricetin improves motor functions and reduces demyelination in vivo.	Cuprizone-treated mice	[96]
	Neuronal damage	Up-regulation of HO-1 contributes to diminish the neuroprotective effects of epigallocatechin-3-gallate in experimental autoimmune encephalomyelitis model.	C57BL/6 mice	[100]
**Ischemia**	Neuroprotection	HO-1 overexpression in hippocampus protects against cerebral I/R activating the BDNF–TrkB–PI3K/Akt signaling pathway.	Sprague-Dawley rats	[77]
		HO-1-mediated neuroprotection is related to enhanced expression of bcl-2, inhibition of nuclear localization of p53 and decreased levels of lipid peroxidation end-products.	HO-1 overexpressing mice	[79]
		HO-1 is required for ischemic preconditioning-induced neuroprotection against brain ischemia.	HO-1 (−/−) Tg mice	[81]
		Pterostilbene administration prevents ischemic brain injury in newborns.	Rat model of neonatal ischemic damage	[80]
**Traumatic brain injury (TBI)**	Neuroprotection	A prolonged glial induction of HO-1 exerts neuroprotection in animal models of traumatic brain injury.	Sprague-Dawley rats	[82]
**Hemorrhage**	Neuroprotection	Selective HO-1 overexpression in astrocytes exerts neuroprotection after intracerebral hemorrhage.	HO-1 overexpressing mice	[83,84]
		Hemin administration reduces BBB damage and improves neurological outcome in experimental models of traumatic and ischemic CNS injury.	Swiss-Webster mice	[85]
		Nrf2/HO-1 up-regulation mediated by t-BHQ administration decreases the development of early brain injury in a subarachnoid hemorrhage model.	Sprague-Dawley rats	[86]
		Nrf2/HO-1 activation mediated by nicotinamide mononucleotide treatment induces neuroprotection after intracerebral hemorrhage.	CD1 mice	[87]
		Fenofibrate reduces neuronal damage of ICH rat brain by increasing HO-1 expression level and decreasing NF-κB expression in PPARα-dependent manner.	ICH rat model, LN-18 glioblastoma cells, rat brain astrocytes	[88]
		HO-1 overexpression and CO generation are necessary to reduce neuronal injury and cognitive dysfunction in a mouse model of subarachnoid hemorrhage.	SAH stroke murine model.	[90]
	Neuroprotection/Neuronal damage	The early up-regulation of HO-1 has a protective role against oxidative stress, whereas late stage overexpression may result in dysfunctions and toxicity in intracerebral hemorrhage.	Sprague–Dawley rats	[89]
**Neuropatic pain**	Neuroprotection	HO-1 up-regulation exerts analgesic effects against neuropathic pain inhibiting spinal microglia activation.	Mouse model of L5 spinal nerve ligation	[102]
		HO-1 induction enhances the antinociceptive effects of morphine via inhibition of microglia activation in painful STZ-induced diabetic neuropathy.	STZ-treated C57BL/6J mice	[103]
**Guillain-Barré syndrome (GBS)**	Neuroprotection	In a mouse model of GBS, treatment with dimethyl fumarate favors macrophages M2 polarization through the up-regulation of Nrf2 and HO-1, preventing inflammation.	Lewis rats	[101]

Abbreviations used in the table: 6-OHDA, 6-hydroxydopamine; AD, Alzheimer’s Disease; Akt, serine/threonine kinase; AMPK, AMP-activated protein kinase; ATR-I, atractylenolide-I; Bcl2, Anti-apoptotic factor B-cell lymphoma 2; BBB, blood brain barrier; BDNF, Brain-derived neurotrophic factor; BVR, biliverdin reductase; CART, cocaine- and amphetamine-regulated transcript; CNS, Central Nervous System; CO, carbon monoxide; DDC, 2′,3′-dihydroxy-4′,6′-dimethoxychalcone; EAE, experimental autoimmune encephalomyelitis; EPO, Erythropoietin; GBS, Guillain-Barré syndrome; HO-1, heme oxygenase-1; I/R, ischemia/reperfusion; ICH, intracerebral hemorrhage; LPS, lipopolysaccharide; MAPK, Mitogen Activated Protein Kinases; MCI, Mild Cognitive Impairment; NB, neuroblastoma; NFκB, nuclear factor kappa light- chain-enhancer of activated B cells; Nrf2, nuclear factor erythroid-derived 2-like 2; PD, Parkinson’s Disease; PI3K, phosphoinositide 3-kinase; PPARα, peroxisome proliferator-activated receptors alpha; SAH, subarachnoid hemorrhage; STZ, streptozotocin; t-BHQ, tert-butylhydroquinone; TrkB, tropomyosinreceptorkinase B.

## Abbreviations

AD	Alzheimer’s Disease
Akt	Serine/threonine kinase
AMPK	AMP-activated protein kinase
AP-1/2	Activator protein 1/2
ARE	Antioxidant Response Elements
ATR-I	Atractylenolide-I
Bach1	BTB domain and CNC homolog 1
BBB	Blood brain barrier
Bcl2	Anti-apoptotic factor B-cell lymphoma 2
BDNF	Brain-derived neurotrophic factor
BVR	Biliverdin reductase
CART	Cocaine- and amphetamine-regulated transcript
CNS	Central Nervous System
CO	Carbon monoxide
CoPPIX	Cobalt protoporphyrin IX
DDC	2′,3′-dihydroxy-4′,6′-dimethoxychalcone
EAE	Experimental autoimmune encephalomyelitis
EPO	Erythropoietin
ERK	Extracellular signal-regulated kinase
Fe^2+^	Free ferrous iron
GBS	Guillain-Barré syndrome
GCLM	Glutamate-Cysteine Ligase Modified subunit
GCLC	Glutamate-Cysteine Ligase Catalitic subunit
GDNF	Glial cell-derived neurotrophic factor
H_2_O_2_	Hydrogen peroxide
HIF1	Hypoxia-inducible factor 1
HMOX1	Heme oxygenase 1 gene
HO	Heme oxygenase
I/R	Ischemia/reperfusion
ICH	Intracerebral hemorrhage
Keap1	Kelch-like ECH-associated protein 1
LPS	Lipopolysaccharide
LXR	Liver-x receptor
MAPK	Mitogen Activated Protein Kinases
MCI	Mild Cognitive Impairment
miRs	microRNAs
MPP^+^	1-methyl-4-phenylpyridine
MPTP	1-methyl-4-phenyl-1,2,3,6-tetrahydropyridine
NB	Neuroblastoma
NF-κB	Nuclear factor kappa light- chain-enhancer of activated B cells
NOS	Nitric oxide synthase
NQO1	NAD(P)H dehydrogenase [quinone] 1
Nrf2	Nuclear factor erythroid-derived 2-like 2
PD	Parkinson’s Disease
PI3K	Phosphoinositide 3-kinase
PPARα	Peroxisome proliferator-activated receptors alpha
ROS	Reactive oxygen species
SAH	Subarachnoid hemorrhage
sGC	Soluble guanylyl cyclase
STZ	Streptozotocin
t-BHQ	Tert-butylhydroquinone
TBI	Traumatic brain injury
TrkB	Tropomyosinreceptorkinase B
6-OHDA	6-hydroxydopamine
